# The vortex formation time to diastolic function relation: assessment of pseudonormalized versus normal filling

**DOI:** 10.1002/phy2.170

**Published:** 2013-11-26

**Authors:** Erina Ghosh, Sándor J Kovács

**Affiliations:** 1 Department of Biomedical Engineering, School of Engineering and Applied Science, Washington University in St LouisSt. Louis, Missouri; 2 Cardiovascular Biophysics Laboratory, Cardiovascular Division, Department of Internal Medicine, School of Medicine, Washington University in St LouisSt. Louis, Missouri

**Keywords:** Diastolic function, tissue Doppler imaging, transmitral flow, vortex formation

## Abstract

In early diastole, the suction pump feature of the left ventricle opens the mitral valve and aspirates atrial blood. The ventricle fills via a blunt profiled cylindrical jet of blood that forms an asymmetric toroidal vortex ring inside the ventricle whose growth has been quantified by the standard (dimensionless) expression for vortex formation time, VFT_standard_ = {transmitral velocity time integral}/{mitral orifice diameter}. It can differentiate between hearts having distinguishable early transmitral (Doppler *E*-wave) filling patterns. An alternative validated expression, VFT_kinematic_ reexpresses VFT_standard_ by incorporating left heart, near “constant-volume pump” physiology thereby revealing VFT_kinematic_'s explicit dependence on maximum rate of longitudinal chamber expansion (*E*′). In this work, we show that VFT_kinematic_ can differentiate between hearts having indistinguishable *E*-wave patterns, such as pseudonormal (PN; 0.75 < *E*/*A* < 1.5 and *E*/*E*′ > 8) versus normal. Thirteen age-matched normal and 12 PN data sets (738 total cardiac cycles), all having normal LVEF, were selected from our Cardiovascular Biophysics Laboratory database. Doppler *E*-, lateral annular *E*′-waves, and *M*-mode data (mitral leaflet separation, chamber dimension) was used to compute VFT_standard_ and VFT_kinematic_. VFT_standard_ did not differentiate between groups (normal [3.58 ± 1.06] vs. PN [4.18 ± 0.79], *P* = 0.13). In comparison, VFT_kinematic_ for normal (3.15 ± 1.28) versus PN (4.75 ± 1.35) yielded *P* = 0.006. Hence, the applicability of VFT_kinematic_ for diastolic function quantitation has been broadened to include analysis of PN filling patterns in age-matched groups.

## Introduction

The ability to quantify diastolic function (DF) quantitatively is crucial in order to properly diagnose heart failure with preserved ejection fraction (HFpEF) or diastolic heart failure (DHF) (Zile and Brutsaert 2002; Gheorghidae and Pang 2009) and to assess the success of therapy. The preferred noninvasive method for DF assessment is Doppler echocardiography and various Doppler indexes are used to quantify DF (Klein and Garcia 2008; Nagueh et al. 2009). Most of these indexes are empiric (based on Doppler echocardiographic waveform features rather than on mechanisms) or correlation based, irrespective of causal relations. Hence, these indexes cannot provide mechanistic insight into the physiology of DF.

A mechanism-based approach for DF quantitation is available and is provided by the parametrized diastolic filling (PDF) formalism (Kovács et al. 1987). Because the heart is a mechanical oscillator, the formalism treats mechanical suction initiated early rapid (Doppler *E*-wave) filling in analogy to the recoil from rest, of a previously displaced, damped simple harmonic oscillator. Model predicted fit to the clinical *E*-wave is excellent and the fitting process specifies three parameters: *k*, the stiffness constant; *c*, the viscoelastic damping/relaxation constant; and *x*_*o*_, the volumetric preload. The PDF formalism has been validated in a broad range of normal and pathophysiologic settings (in humans and animals). The PDF parameters and indexes derived from them have been rigorously shown to have direct clinical relevance (Dent et al. 2001; Lisauskas et al. 2001a,b; Riordan and Kovács 2006; Shmuylovich and Kovács 2006). The PDF formalism has been automated (Hall and Kovács 1994; Hall et al. 1998) and solves the “inverse problem of diastole” (Hall and Kovács 1993) by providing a unique set of PDF parameters for each analyzed *E*-wave.

An alternate approach for DF characterization utilizes fluid mechanics. The left ventricle (LV) fills by aspirating atrial blood which forms an asymmetric toroidal (doughnut-shaped) vortex as it curls around the mitral leaflet tips (Hong et al. 2008). The vortex ring expands as the ventricle fills and the outer boundary of the vortex rinses the highly trabeculated endocardium preventing thrombus formation while concomitantly facilitating mitral leaflet coaptation during diastasis (Ghosh and Kovács 2011). The pattern of flow and vortex formation is affected by cardiac dysfunction and has been previously characterized via echocardiography using vortex formation time (VFT).

Gharib et al. (2006) used Doppler *E*-wave data to calculate VFT_standard_ in subjects with normal LVEF and normal *E*-wave patterns and subjects with dilated cardiomyopathy and abnormal *E*-wave patterns. They found that subjects with normal *E*-wave patterns had a normal range of values (3.5–5.5), whereas subjects with dilated cardiomyopathy had lower VFT_standard_.

We have previously derived and validated a complementary method of calculating VFT (VFT_kinematic_) (Ghosh et al. 2009, 2010) involving the PDF formalism (Kovács et al. 1987) (See Appendix A for details). Our derivation made use of the near constant-volume physiologic attribute of the left heart (Bowman and Kovács 2003) that provides the algebraic relationship between effective mitral orifice area (diameter) and longitudinal annular tissue motion (*E*′). Our results demonstrated very good correlation between VFT_kinematic_ and (*E*/*E*′)^3/2^, an established echocardiographic index of DF (Nagueh et al. 1997; Ghosh et al. 2010).

In this work, we test the hypothesis that VFT_kinematic_ can distinguish between normal and diastolic dysfunction (pseudonormal [PN] filling) states where both groups are age matched and have indistinguishable, normal *E*-wave patterns. To test our hypothesis we analyzed 738 beats and computed VFT_kinematic_ and VFT_standard_ in 25 subjects and performed an intergroup comparison.

## Methods

### Subject selection criteria

Echocardiographic data from 25 subjects were selected from the Cardiovascular Biophysics Laboratory database. Prior to data acquisition, subjects provided signed, informed consent for participation in accordance with the Institutional Review Board (Human Research Protection Office) at Washington University School of Medicine. The inclusion criteria were as follows: normal sinus rhythm, absence of valvular abnormalities and the absence of wall-motion abnormalities or bundle branch block on the ECG, normal LVEF, normal valvular function, and clearly identifiable *E*- and A-waves and *E*′-waves. In addition, all subjects also had Doppler *M*-mode images of the mitral leaflet motion recorded in the parasternal view.

We dichotomized subjects into normal and PN groups, according to American Society of Echocardiography (ASE) (Nagueh et al. 2009) criteria. In both groups 0.75 < *E*/A < 1.5, in the normal group, lateral *E*′_peak_ velocity was >10 cm/sec and *E*/*E*′ < 8 and in the PN group, lateral *E*′_peak_ velocity was reduced (<10 cm/sec) resulting in *E*/*E*′ > 8. All subjects (both groups) had normal LV ejection fraction (>50%) and either normal coronary anatomy or insignificant (<50%) coronary artery narrowing. Because diastolic filling patterns (Klein et al. 1994) and VFT_standard_ depend on age (Gharib et al. 2006), we specifically age matched the groups so that age could not be a distinguishing metric.

### Doppler echocardiography

**Figure 1:**
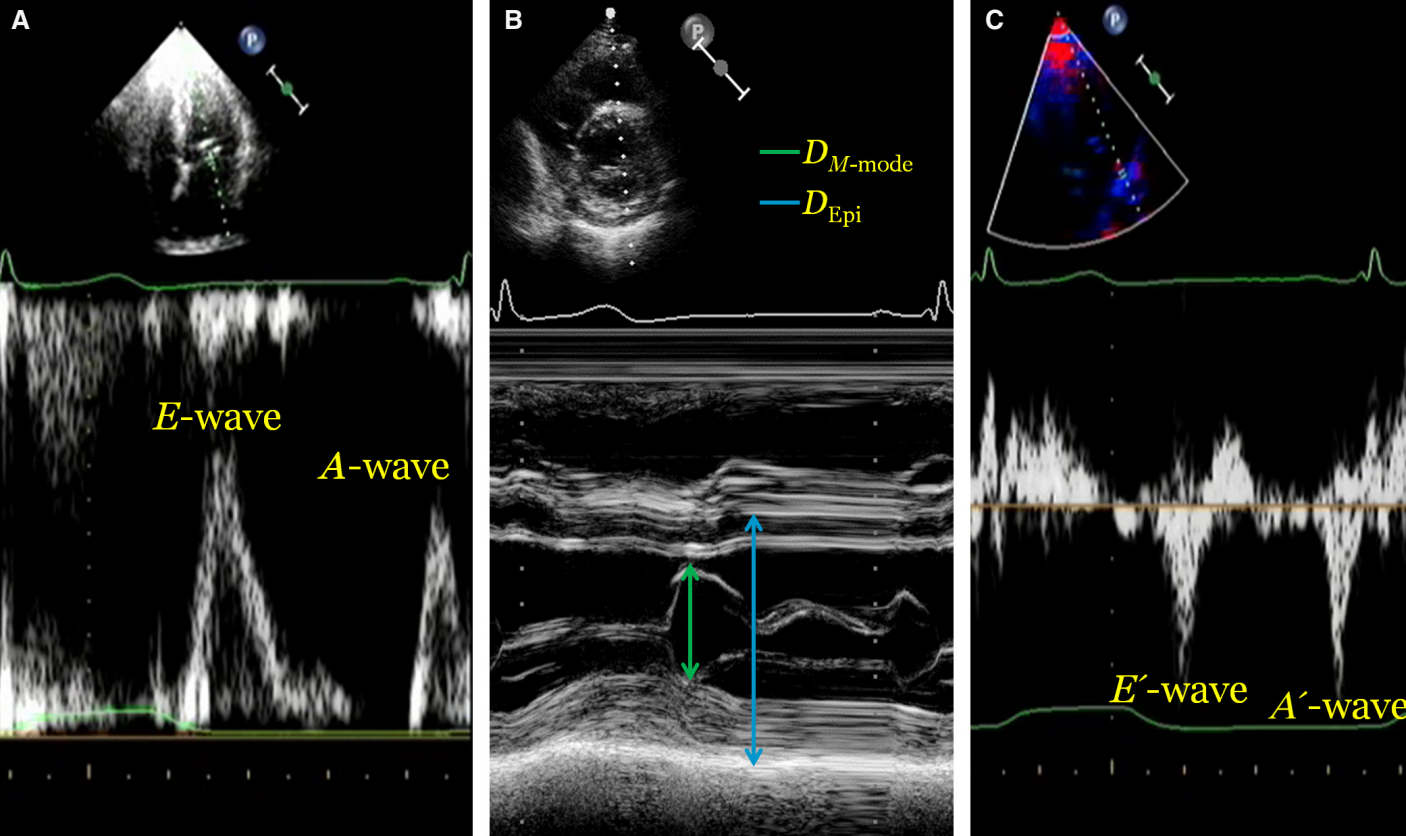
(A) Typical Doppler transmitral flow image showing one cardiac cycle with Doppler *E*- and *A*-wave marked. (B) Typical parasternal *M*-mode image with peak mitral leaflet separation (*D*_*M*__-mode_) in green and epicardial diameter (*D*_epi_) in blue. (C) Typical tissue Doppler, lateral mitral annulus motion image with Doppler *E′*- and *A′*-wave marked. See text for details.

Our data acquisition method has been previously detailed (Shmuylovich and Kovács 2006; Ghosh et al. 2010). Briefly, echocardiography was performed in accordance with published ASE criteria (Nagueh et al. 2009). Immediately before catheterization, patients were imaged in a supine position using a Philips iE33 system (Best, the Netherlands). Two-dimensional images in apical two- and four-chamber views were obtained with the sample volume gated at 1.5–2.5 mm directed between the tips of the mitral valve leaflets and orthogonal to the mitral valve plane (to minimize misalignment effects). The wall filter was set at 125 or 250 Hz, the baseline adjusted to take advantage of the full height of the display and the velocity scale adjusted to exploit the dynamic range of the output without aliasing. The four-chamber view was used to record Doppler *E*-waves and tissue Doppler *E*′-waves (Fig. 1A and C). For *E*′-wave recording the lateral aspect of mitral annulus was selected because recent studies have shown that in patients with normal ejection fraction, lateral *E*′ has the best correlation with LV filling pressures and invasive indices of LV stiffness (Kasner et al. 2007). Doppler *M*-mode images recorded in the parasternal short-axis view (as shown in Fig. 1B) were used to determine mitral leaflet separation for effective orifice diameter computation following the European Association of Echocardiography (EAE)/ASE guidelines (Baumgartner et al. 2009).

### Doppler data analysis

The transmitral Doppler waveforms and the tissue Doppler *E*′-waves were analyzed using two methods: (1) the conventional method and (2) the PDF formalism, using best *E*-wave contour fit criteria in computing the PDF (*x*_*o*_, *c*, *k*) parameters. In the conventional method, the *E*-wave is approximated as a triangle such that its height is peak velocity (*E*_peak_) and its base is the duration (*E*_dur_). The same method is used to analyze Doppler *E*′-wave and determine *E*′_peak_ and *E*′_dur_.

The second method of *E*-wave analysis employs the PDF formalism (Kovács et al. 1987) which solves the “inverse-problem of diastole” (Hall and Kovács 1993) (See Appendix A). The method of PDF parameter determination has been automated and detailed previously (Hall et al. 1998; Riordan and Kovács 2006) and has an interobserver variability of 8% (Boskovski et al. 2008).

The peak mitral leaflet separation was calculated from *M*-mode images in accordance with (Gharib et al. 2006). The epicardial dimension *D*_epi_ was also measured from the same *M*-mode image using the method described by Foppa et al. (2005). Figure 1B shows a typical *M*-mode image with the diameters marked.

### Calculation of VFT

The method of calculating VFT_kinematic_ and VFT_standard_ has been previously detailed (Ghosh et al. 2010). Briefly, VFT_standard_ is defined as the *E*-wave area (triangle method) divided by the maximum mitral leaflet separation.


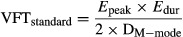
(1)

Using equation (1), VFT_standard_ is calculated for each *E*-wave using the needed parameters determined from the conventional method described in the previous section. The diameter is measured from parasternal *M*-mode image as shown in Fig. 1B. We have previously shown (Ghosh et al. 2010) that the *E*-wave area calculated via the triangle method is equivalent to *E*-wave area calculated by integrating the PDF fit curvilinear contour of the *E*-wave.

VFT_kinematic_ also uses the same *E*-wave area divided by the orifice diameter expression. However, *E*-wave area is calculated using the PDF formalism derived curvilinear fit and the flow orifice diameter is derived by incorporating the near constant volume attribute of the left heart (Bowman and Kovács 2003; Ghosh et al. 2010). The previously derived and validated expression for VFT_kinematic_ is as follows:


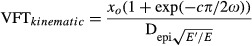
(2)

where *D*_epi_ is a constant, the epicardial diameter measured from *M*-mode images (blue line in Fig. 1B), *c*, and *x*_*o*_ are PDF parameters, and *ω* is the angular frequency of a full oscillation, the initial half of which corresponds to the *E*-wave. These are obtained by analyzing the Doppler *E*-wave (Fig. 1A) using the PDF formalism. In the denominator, *E*′_peak_ and *E*_peak_ are obtained as in the conventional analysis.

### Numerical methods and statistical analyses

Images were recorded in DICOM format and converted into bitmap images using a custom MATLAB program (MATLAB 6.0 MathWorks, Natick, MA). Another MATLAB script was written to compute the conventional triangle fit to Doppler *E*- and *E*′-waves. The PDF parameters were computed using an error-minimizing algorithm (Levenberg–Marquardt algorithm) as detailed briefly in Appendix (A) and fully in previous work (Ghosh et al. 2010). VFT_kinematic_ and VFT_standard_ values were computed for each beat using a custom MATLAB program. VFT_standard_ was calculated for each *E*-wave using equation 1 and VFT_kinematic_ was calculated by matching *E*- and *E*′-waves with close R-R intervals (<10 msec difference). For each subject, the values of VFT were averaged for all the beats. Student's *t*-test (two tailed) was used to determine the significance of difference between the two groups. *P* < 0.05 was considered statistically significant. Regression analysis was performed to validate the relationship between both the VFTs and (*E*/*E*′)^3/2^. The average values were correlated and Pearson's product moment correlation coefficient (*R*^2^) for each linear regression was determined.

## Results

A total of 738 beats from 25 subjects were analyzed (average 30 beats/subject). Table 1 provides group demographics involving 12 men and 13 women. Nine men and four women were in the normal group and three men and nine women were in the PN group. The groups were age matched (normal = 59 years, range: 49–73 years and PN = 61 years, range: 47–78 years, *P* = 0.59). Body surface area and weight of the two groups were not significantly different. The left ventricular end diastolic pressure (LVEDP) of 14 mmHg for normal and 16 mmHg for PN (*P* = 0.12) did not differ between groups. The EF of all subjects was normal. Although normal, the EF of the PN group was higher (74%) than the EF of the normal group (67%). The mean resting HR was slightly higher in the PN group (71 bpm) than normal (61 bpm).

**Table 1:** Subject demographics showing data for the two groups and the *P*-value (Column 4)

Parameter	Normal (*n* = 13)	PN (*n* = 12)	*P*-value
Age (years)	59 (9)	61 (12)	NS 0.59
Gender	9M, 4F	3M, 9F	
Weight (lbs)	177 (28)	189 (54)	NS 0.49
Height (cm)	173 (8)	160 (14)	0.014
BSA (m^2^)	1.95 (0.2)	1.96 (0.3)	NS 0.96
EDP (mm Hg)	14 (3)	16 (4)	NS 0.12
Ejection Fraction (%)	67 (9)	74 (7)	0.03
Mean HR (bpm)	61 (8)	71 (13)	0.04
Beats Analyzed	26 (18)	33 (21)	NS 0.41

*P* < 0.05 is considered statistically significant. The standard deviation values are in parenthesis.

Echo parameters: Table 2 gives the group average values of echocardiographic parameters and PDF parameters. There was no statistical difference between Doppler *E*-wave parameters (conventional and PDF) between the two groups. The *E*_peak_ was 70 cm/sec for normal and was 72 cm/sec for PN. The *E*_dur_, DT, and the velocity time integral (VTI) were not different between the groups. The *A*_peak_ velocity was higher in PN group (82 cm/sec) than normal (55 cm/sec). Although *E*/*A* was statistically different between the two groups, the value for both the groups was in the normal range (0.75–1.5) for the age range (Nagueh et al. 2009). In accordance with convention for PN patterns, *E*′_peak_ velocity was significantly lower in the PN group (normal = 14 cm/sec vs. PN = 7 cm/sec, *P* < 0.001), however *E*′_dur_ was not different. In accordance with PN pattern criteria *E*/*E*′ was significantly higher than control (normal = 5.3 vs. PN = 10.7, *P* < 0.001).

**Table 2:** *E*- and *E′*-wave parameters

Parameters	Normal (*n* = 13)	PN (*n* = 12)	*P*-values
*E*_peak_ (cm/sec)	70 (16)	72 (13)	NS 0.76
*E*_dur_ (msec)	294 (26)	299 (31)	NS 0.66
DT (msec)	200 (28)	203 (32)	NS 0.81
*A*_peak_ (cm/sec)	55 (11)	82 (16)	<0.001
*E*/*A*	1.3 (0.3)	0.9 (0.2)	<0.001
*E′*_peak_ (cm/sec)	14 (5)	7 (1)	<0.001
*E*/*E′*	5.3 (1.4)	10.4 (2.0)	<0.001
VTI (cm)	10 (3)	11 (2)	NS 0.67
PDF parameters			
*k*	263 (53)	254 (53)	NS 0.68
*c*	23 (6)	25 (7)	NS 0.43
*x*_*o*_	9.2 (2.1)	10.3 (2.3)	NS 0.21

The mean values for each group are listed along with *P*-value. *P* < 0.05 denotes statistical significance. Parentheses denote standard deviations. VTI, velocity time integral; PDF, parametrized diastolic filling.

**Figure 2:**
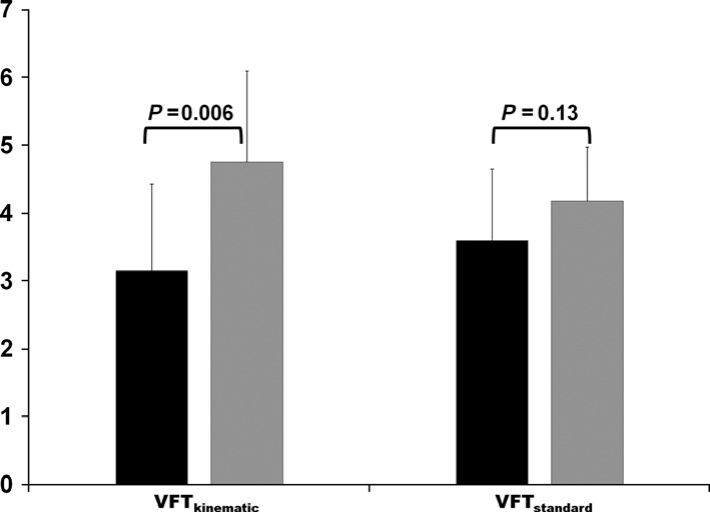
Comparing VFT_kinematic_ and VFT_standard_ in the normal and PN groups. The bars on the left compare the value of VFT_kinematic_ in normal (dark gray) and PN (light gray) subjects. The bars on the right show the same comparison for VFT_standard_ values. The error bars represent the standard deviation for the four values. The *P*-values of the intergroup comparison for each VFT value is given. See text for details. VFT, vortex formation time; PN, pseudonormal.

VFT values: The mean VFT_kinematic_ for the normal group was 3.15 and for the PN group it was 4.75. VFT_standard_ for the normal group was 3.59 and for PN it was 4.18. The differences in VFT_kinematic_ were statistically significant (*P* = 0.006), whereas the differences in VFT_standard_ were not (*P* = 0.13). This is shown in Figure 2. Table 3 lists the value of VFT_kinematic_ and VFT_standard_ for all subjects.

**Table 3:** VFT_kinematic_ and VFT_standard_ for all 25 subjects with associated component values

Subject No.	VFT_kinematic_	VFT_standard_	Epicardial diameter (cm)	Maximum leaflet Separation (cm)	*E*_peak_ (m/sec)	*E′*_peak_ (m/sec)	*E*/*E′*
1	2.6	3.3	7.0	2.9	0.57	0.15	3.7
2	1.5	2.2	10	3.6	0.50	0.11	4.5
3	1.8	2.0	7.8	3.4	0.52	0.11	4.7
4	1.9	3.3	7.9	2.6	0.53	0.10	5.1
5	2.2	3.7	7.8	2.4	0.65	0.13	5.1
6	3.0	3.1	6.5	2.8	0.66	0.12	5.6
7	4.6	4.5	6.1	2.8	0.90	0.16	5.7
8	4.1	3.9	7.3	3.6	0.86	0.12	7.1
9	5.8	6.3	6.4	2.5	1.02	0.14	7.1
10	3.3	3.8	6.5	2.7	0.70	0.13	5.3
11	3.9	3.8	6.1	2.5	0.71	0.14	5.5
12	2.2	3.2	6.1	3.1	0.67	0.29	2.3
13	4.1	3.5	7.6	3.3	0.84	0.11	7.7
14	3.3	3.8	8.7	2.5	0.69	0.08	8.6
15	4.5	4.2	6.4	2.3	0.62	0.07	8.6
16	1.9	2.8	11	3.0	0.55	0.07	8.5
17	4.5	3.9	5.9	2.4	0.74	0.08	9.2
18	6.7	5.2	6.4	2.5	0.76	0.07	11.4
19	6.7	5.7	6.8	2.4	0.92	0.07	12.7
20	6.0	4.6	6.9	2.5	0.87	0.07	13.3
21	4.5	3.4	6.3	2.5	0.61	0.05	13.6
22	4.9	3.9	6.6	2.7	0.68	0.06	11.8
23	5.3	4.3	6.5	2.7	0.81	0.09	9.1
24	4.6	4.7	9.2	3.0	0.89	0.09	9.3
25	4.0	3.7	6.9	2.5	0.52	0.06	9.1

Subjects 1–13 are the normal group. Subjects 14–25 are the PN group. VFT, vortex formation time; PN, pseudonormal.

## Discussion

Vortex ring formation in the LV manifests nature's solution to the “atrium to ventricle mass transfer problem,” while maintaining efficient filling by helping to preserve the momentum of blood flow and by optimally aligning streamlines toward the outflow tract (Gharib et al. 1998; Mohseni and Gharib 1998; Krueger and Gharib 2003; Pedrizzetti and Domechini 2005; Pasipoularides 2010; Töger et al. 2012). The fluid mechanics of vortex formation has been extensively studied and adapted to quantify LV filling dynamics (Kheradvar et al. 2007). Studying the physiology of filling through VFT provides a novel way to assess DF. We previously derived and validated an alternate expression for VFT which includes explicit *E*/*E*′ dependence (Ghosh et al. 2010). In this study, we build on that foundation by extending the physiologic realm of VFT applicability by testing the ability of the alternate VFT expression to differentiate between age-matched controls and subjects with PN filling (characterized by essentially indistinguishable *E*-wave patterns). Our goal in this study was to demonstrate the advantage of using a physiology based expression of VFT to quantify diastolic filling, rather than enhancing the understanding of LV vortex dynamics.

### Previous studies

To facilitate clinical application Gharib et al. (2006) proposed a simplified definition for VFT described above (eq. 1). Using this expression they showed that VFT_standard_ had an optimal range of values. Other studies (Lee et al. 2007; Jiamsripong et al. 2009a,b; Kheradvar et al. 2012) have also shown that VFT_standard_ can differentiate between normal *E*-wave patterns and selected pathologic *E*-wave patterns.

VFT_standard_ uses only Doppler *E*-wave features and mitral orifice diameter at a single time point during filling. However, there is a causal relationship between chamber (motion) kinematics and fluid (motion), hence VFT can be shown to depend on the kinematic attributes of the LV (Ghosh et al. 2009, 2010). Vortex formation is affected by how the LV accommodates volume, which includes longitudinal (characterized by the tissue Doppler *E*′-wave) and radial filling volume components. By incorporating near constant volume physiology, VFT_kinematic_ explicitly takes into account longitudinal motion at the level of the annulus. As a consequence, we previously (Ghosh et al. 2010, Appendix B) demonstrated that in an idealized (cylindrical) LV aspirating essentially constant *E*-wave volumes (in reality <10% variation among subjects), VFT is proportional to (*E*/*E*′)^3/2^. We achieved a significant correlation of VFT_kinematic_ versus (*E*/*E*′)^3/2^ while VFT_standard_ failed to achieve a significant correlation versus (*E*/*E*′)^3/2^.

**Figure 3:**
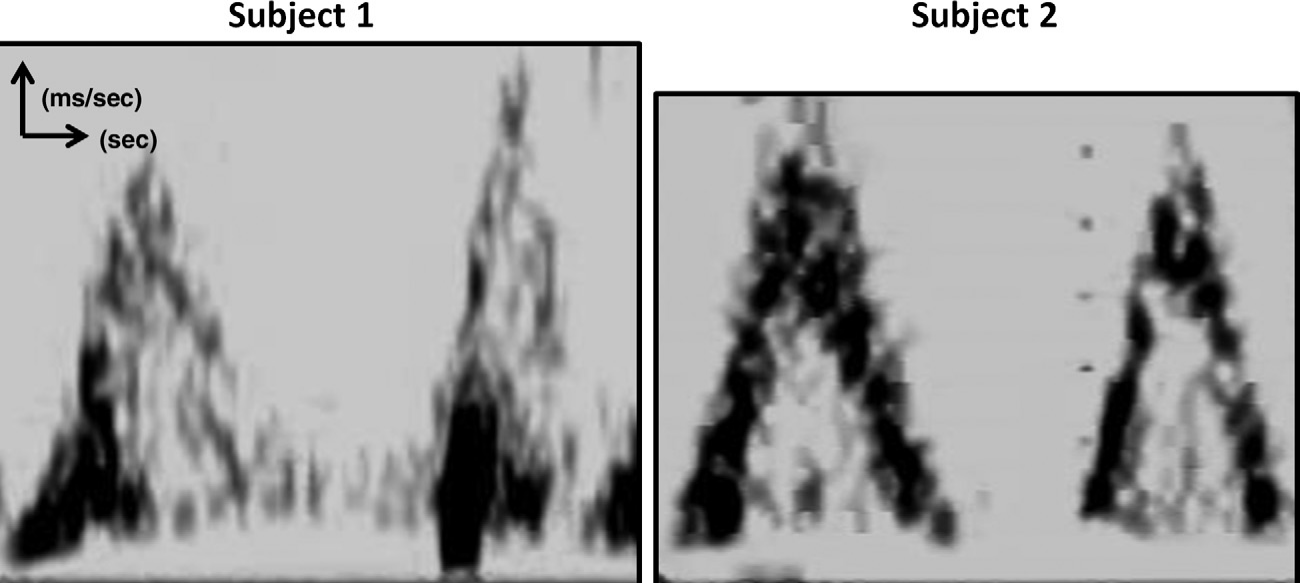
Comparing the ability of VFT_kinematic_ and VFT_standard_ to differentiate between two similar Doppler *E*-waves. The *E*-wave image on the left (marked Subject 1) is from Subject 5 (Table 3; normal group). The image on the right (marked Subject 2) is from Subject 17 (PN group). VFT_standard_ can not differentiate between these two waves while VFT_kinematic_ can differentiate between them. See text and Table 4 for details. VFT, vortex formation time.

Recently, Kheradvar et al. (2012) calculated VFT_standard_ in four groups of subjects grouped according to *E*-wave patterns: normal, impaired relaxation, PN relaxation, and restrictive filling. They showed that VFT_standard_ was significantly different among the four groups, demonstrating the ability of VFT_standard_ to correlate with altered transmitral filling patterns associated with increasing dysfunction. However, the results were confounded by age, since the age of the normal subject group was less than half of the ages of the other three groups (Normals = 28 years vs. DD = 63 years). Previous work by Gharib et al. (2006) (Fig. 3 of referenced paper) has shown that VFT_standard_ decreases substantially with age. In light of this dependence of VFT_standard_ on age, the reported differences in VFT_standard_ in (Kheradvar et al. 2012) are confounded by age.

### Identifying PN filling

In moderate DD, characterized by PN filling, the transmitral *E*- and *A*-wave shapes have the same characteristics as *E*- and *A*-waves in normal subjects. An example is shown in Figure 3 which shows *E*-waves from two subjects. The *E*-waves are indistinguishable using conventional metrics such as peak velocity, duration, VTI, and E/A (Table 4). However, they have different peak *E*′. VFT_standard_ cannot distinguish between these two subjects because it depends only on *E*-wave features. However, VFT_kinematic_ can differentiate between them because it incorporates *E*′. The PN pattern of DD has impaired longitudinal motion, elevated LAP while maintaining a normal *E*-wave shape.

**Table 4:** Features of two Doppler *E*-waves shown in Figure 3 with associated VFT values

Parameter	Subject 1	Subject 2
*E*_peak_ (cm/sec)	60	60
*E*_dur_ (msec)	217	217
HR (bpm)	68	61
*E′*_peak_ (cm/sec)	12	6.6
VFT_standard_ (dimensionless)	2.71	2.69
VFT_kinematic_ (dimensionless)	1.75	3.16

The two *E*-waves are indistinguishable in terms of mean *E*_peak_, *E*_dur_, and *E*/*A*. HR and VFT_standard_ values.

For this study, we selected subjects whose echo data satisfy the established criteria for the PN pattern by having *E*-waves similar to the normal group, normal LV function (EF > 50%) but impaired longitudinal (*E*′) motion. The PN group had *E*′_peak_ < 10 cm/sec and *E*/*E*′> 8. Previous studies (Nagueh et al. 1997; Mohseni and Gharib 1998) have used *E*/*E*′ to estimate filling pressures and to assess DF in subjects with normal EF. We used their cutoff value of 8 to dichotomize into PN versus normal groups. In addition, to ensure that the difference in the *E*/*E*′ ratio is not due to reduced *E*_peak_ velocity, we required lateral *E*′_peak_ < 10 cm/sec for the PN group. Previous studies have shown (Klein et al. 1994; Hill and Palma 2005) that for the age range in this study, normal *E*′_peak_ velocities have a mean value of ∼11 cm/sec. As DF becomes impaired with age (Klein et al. 1994; Ommen and Nishimura 2003; Hill and Palma 2005; Nagueh et al. 2009), we age matched the groups. The Doppler *E*-wave attributes in both of the groups studied were similar (Table 2). The two groups differed in the Doppler *E*′_peak_ representing the peak longitudinal volume accommodation rate.

**Figure 4:**
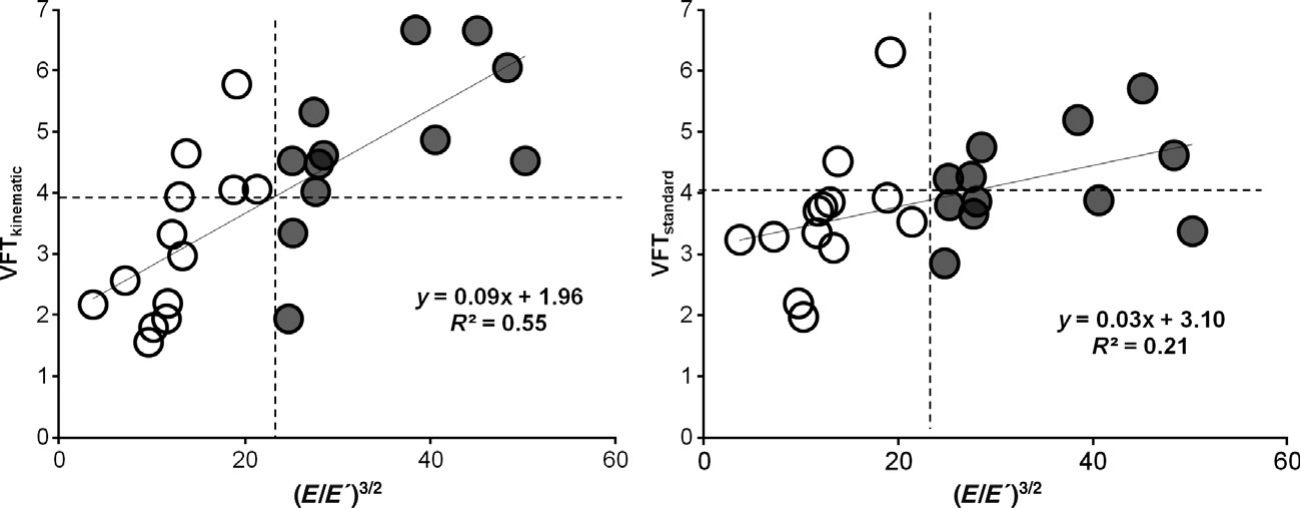
Comparing VFT_kinematic_ and VFT_standard_ to *E*/*E′* ^3/2^. Open circles represent subjects in the normal group and closed subjects represent subjects in the PN group. (A) Correlation between VFT_kinematic_ and *E*/*E′* ^3/2^. The two ratios are well correlated (*R*^2^ = 0.55) and nine of 13 subjects in the normal group have VFT_kinematic_ < 4. Eleven of 13 subjects in PN group have VFT_kinematic_ > 4. (B) Correlation between VFT_standard_ and *E*/*E′* ^3/2^. The two ratios are not well correlated and no clear cutoff value for VFT_standard_ exists. Ten of 12 normal subjects had VFT_standard_ < 4 and six of 12 PN subjects had VFT_standard_ > 4. See text for details. VFT, vortex formation time; PN, pseudonormal.

In our previous work, we derived and demonstrated the relationship between VFT and (*E*/*E*′)^3/2^ (Appendix B, Ghosh et al. 2010). Using the constant volume attribute (Bowman and Kovács 2003) we demonstrated that for the same *E*-wave volume, VFT is inversely proportional to *D*^3^, where *D* is effective orifice diameter. Since *D* is proportional to √(*E*^′^/*E*), VFT is proportional to (*E/E′*)^3/2^. As VFT_kinematic_ incorporates the constant volume attribute, it provides a better correlation with (*E/E′*)^3/2^ than VFT_standard_. Figure 4 compares VFT_kinematic_ and VFT_standard_ to (*E*/*E′*)^3/2^. Although the relationship between VFT and (*E*/*E′*)^3/2^ is based on approximations (assuming that the LV is a cylinder with no epicardial radial expansion and *E*-wave volumes among subjects remain essentially constant), VFT_kinematic_ had a good correlation with (*E*/*E′*)^3/2^ (*R*^2^ = 0.55). In most of the normal subjects, (open circles; 9 of 13) VFT_kinematic_ < 4 and in most PN subjects (10 of 12), VFT_kinematic_ > 4 (Fig 4A). VFT_standard_, however, had a poor correlation with (*E*/*E′*)^3/2^ (Fig. 4B). There is no clear value of VFT_standard_ which can differentiate between the groups. This is restated in Table 3, which lists the mean values of VFT_kinematic_ and VFT_standard_ for all subjects. Hence, VFT_kinematic_ not only correlates better with *E*/*E′*, an established DF index (Nagueh et al. 1997) but is also able to better dichotomize between groups. Our study builds upon our previous work and expands the applicability of VFT_kinematic_.

### Physiological and clinical significance

Traditionally, VFT has been computed in vitro using a piston-cylinder arrangement where the fluid exited the nozzle and formed vortices in a larger (essentially unbounded) space (Dabiri and Gharib 2005; Kheradvar and Gharib 2008). In the LV, the fluid (blood) is aspirated through the mitral orifice into a smaller (bounded) space (LV chamber) where expanding vortex ring dimension is comparable to expanding chamber dimension (Töger et al. 2012). In this scenario, the rate of chamber expansion plays an important role in vortex formation by actually generating the space for the vortex ring to expand into while providing the energy to generate the flow (since early transmitral filling is powered by the LV chamber recoil/suction). Optimal vortex formation occurs when vortex ring size and growth is synchronized with chamber size and chamber expansion (Kheradvar et al. 2007; Kheradvar and Gharib 2008). A mismatch between these two dynamic (tissue motion and fluid motion) attributes, as seen in enlarged chambers with a low LVEF, results in suboptimal vortex formation, indicative of less efficient utilization of recoil energy and associated mass transfer (Töger et al. 2012). When the rate of chamber expansion is slower than vortex ring growth, (as in an enlarged chamber) the kinetic energy of blood is lost and fluid momentum is not directed toward the outflow track for optimal subsequent ejection. This has been shown by Carlhäll and Bolger (2010) who found that in normal hearts most of the ejected blood volume entered the LV in the same beat, whereas in subjects with moderate heart failure, the volume of blood entering and exiting the LV in the same beat is decreased. Consequently, the LV has to provide the energy to eject the stagnant blood thereby making the filling and ejection process less efficient. Tissue Doppler *E′* velocity has been shown to be reduced in DD. A slower longitudinal chamber expansion rate corresponding to lower *E′*_peak_ or a higher *E*/*E′* ratio is a correlate of higher filling pressures (Nagueh et al. 1997; Lisauskas et al. 2001b). In terms of energetics, this means that the ventricle does more work to fill to the same volume.

Thus, by explicitly including chamber expansion (*E′*), VFT_kinematic_ includes the relation of filling physiology to fluid mechanics. The original expression for VFT = v*t/D = L/D, where *v* is flow velocity and *t* is time of flow duration, facilitates inclusion of the near constant-volume attribute of the left heart. Specifically, near constant volume means that left atrial and ventricular volumes reciprocate as a result of ascent and descent of the mitral annulus and LV wall thickening and thinning throughout the cardiac cycle, hence the LA–LV summed total (epicardial) volume is essentially constant. This defines the algebraic (volume conserving) relation among LV wall thinning, transmitral flow (*E*-wave), mitral annular velocity (*E′*-wave) flow orifice diameter (*D*), and constant epicardial dimension.

Another issue that affects the calculation of both VFT_kinematic_ and VFT_standard_ is the question of relative versus absolute velocity. In deriving VFT and expressing it as v*t/D = L/D, the implicit assumption is that the flow velocity is measured relative to a stationary orifice. In the heart, however, the ultrasonic transducer defines the origin of the coordinate system relative to which transmitral flow (*E*-wave) is measured. Whereas the blood enters the ventricle at the velocity of the *E*-wave, the orifice (mitral annulus and valve) is moving in the opposite direction (relative to the transducer) at the velocity of the *E′*-wave. Hence, relative to the orifice itself, the blood is moving at velocity *E* + *E′*. This has the effect of increasing the VTI relative to the original value by about 10% for normals and by about 5% for PNs (because *E′* VTI is statistically significantly lower in the PN group). If we recalculate group differences correcting for the motion of the annulus (i.e. using *E* + *E′*) for VTI rather than just *E*, our results remain unaltered, we again find that statistically significant difference between VFT_kinematic_ *P* = 0.011 versus VFT_standard_ *P* = 0.26 is maintained. Recall, prior to the *E* + *E′* “correction” the differences in VFT_kinematic_ were P = 0.006 whereas for VFT_standard_ it was *P* = 0.13.

DHF or HFpEF is present in ∼50% of patients admitted to hospitals with heart failure. The mortality rate of HFpEF is slightly lower than the subjects with reduced ejection fraction (Bhatia et al. 2006; Owan et al. 2006). Echocardiography is the preferred method for noninvasive diagnosis and grading of HF (Nagueh et al. 2009). Previous studies have shown (Møller et al. 2000) that in patients after a myocardial infarction, PN filling was an independent predictor of mortality. Subjects with PN filling had higher mortality than patients with normal filling or impaired relaxation. A specific therapeutic approach for selective treatment of HFpEF still eludes us (Schwartzenberg et al. 2012). Hence, the availability of indexes that incorporate fluid mechanics attributes of filling and can differentiate PN from normal filling is of value.

Although the fact that the PN and normal groups in this sample can be easily differentiated using conventional metrics (*E′*, EF, *A*_peak_) may be initially viewed as a limitation to ultimate clinical utility, the fact that VFT_kinematic_ is derived from basic principles of fluid mechanics, and incorporates near constant volume (Bowman and Kovács 2003) and suction pump physiology of the LV while providing explicit time-dependent expressions for its components enhances its ultimate value in merging fluid mechanics–based analysis of DF.

Currently, fluid streamline imaging is in its infancy, but the rapid advancement of noninvasive imaging technology will lead to streamline information application in multiple modalities such as echo, MRI, and CT (recall the advances in echo from 1*D* (*M*-mode) to 2*D* to Doppler, to color-Doppler to 3*D*) (Sengupta et al. 2012). The characterization of the relation between VFT and physiology will become increasingly important and will likely lead to understanding of new relationships between flow and chamber function. Currently, this form of *VFT is* a crude (lumped parameter) metric of the (global) wall motion (DF) to streamline generation relation. When viewed in this context, our work is the first step in incorporating the physiology and merging the technology of streamline imaging and characterization and global DF. We anticipate full, high spatial, and temporal resolution, 3D streamline information availability as the technology advances.

### Limitations

Limitations arise from the definition (eqs. 1 and 2) and the echocardiographic data used to calculate them. The various assumptions and limitations in calculating VFT_kinematic_ and VFT_standard_ have been discussed previously (Ghosh et al. 2010). The peak mitral leaflet separation is calculated from *M*-mode images in parasternal short-axis view that can be affected by a poor acoustic window, transducer position, and the angulation relative to LV long axis. To mitigate these effects we made multiple measurements of the diameter whenever possible and averaged over measured values. We also measure the diameter from one-dimensional *M*-mode measurement which might result in error if the orifice is not round or if the measurements were not made at the center of the valve plane. Because this is an accepted limitation of *M*-mode imaging, we took care to calculate VFT using the method previously used in (Gharib et al. 2006; Ghosh et al. 2010).

The calculation of VFT_kinematic_ should use simultaneous *E*- and *E′*-waves. Accordingly we matched *E*- and *E′*-waves by selecting beats with R-R intervals within ±10 msec of each other. All subjects had a diastatic interval, hence the minor R-R differences affect only diastasis duration.

The number of subjects in the groups is constrained by the number of subjects in the Cardiovascular Biophysics Laboratory database who satisfy the inclusion criteria. The large number of beats analyzed (738) mitigates this limitation to an acceptable degree and provides adequate power for our statistics-based conclusions. However, clinical use awaits studies having a larger sample size. We have specifically chosen groups with normal ejection fraction and clinically indistinguishable *E*-waves to demonstrate the advantage of VFT_kinematic_ over VFT_standard_ to detect differences. Gharib et al. (2006) and Kheradvar et al. (2012) have shown that VFT in subjects with reduced ejection fraction is different from subjects with a normal ejection fraction.

Another potential limitation of the study is gender distribution. While the overall number of men and women studied was nearly equal, they were unequally divided into the two groups. The normal group had nine men of 13, whereas the PN group had nine women of 12. The gender-based differences in Doppler echocardiographic indexes are well documented (Sadaniantz et al. 1997; Bella et al. 2002). Claessens et al. (2011) computed conventional and PDF *E*-wave parameters (*x*_*o*_, *c*, and *k*) and found that in 1606 age-matched subjects (862 females/744 males) the PDF parameters *x*_*o*_, *c*, and *k* were significantly lower in males as compared to females. Other studies (Park et al. 2007) have reported no significant gender-based differences in *E*/*A*, *E*-wave DT, and *E*/*E′* (lateral or septal). However, the effect of gender on VFT has not been studied. In this study, we computed the gender-based group average for VFT_kinematic_ and VFT_standard_ and found that these values were not statistically different between men and women. Hence, in our study, gender did not affect the difference in VFT_kinematic_ between normal and PN groups. However, to reliably assess gender-related issues larger groups will need to be studied. Nonetheless since HFpEF is more prevalent in women (Bhatia et al. 2006), the PN subset in our study approximates that population.

## Conclusion

VFT defined as VFT_standard_ = {*E*-wave VTI}/{mitral orifice diameter} has been shown to differentiate between diastolic dysfunction groups defined by distinguishable *E*-wave patterns and decreasing LVEF. To extend the clinical realm of VFT applicability, we tested the hypothesis that VFT can also differentiate between age-matched groups having PN patterns versus age-matched controls, with normal EF and indistinguishable *E*-waves. We compared VFT_kinematic_ and VFT_standard_ and found that VFT_kinematic_ could differentiate between groups. Because VFT_kinematic_ incorporates *E′* it expands the clinical realm of VFT applicability.

## Appendix A

### The parametrized diastolic filling (PDF) formalism

A causal, kinematic, physiologic mechanism-based model of DF – the parametrized diastolic filling (PDF) formalism (Kovács et al. 1987) – has been previously derived and validated. The PDF formalism models suction-initiated filling in analogy to the motion of a damped, simple harmonic oscillator (SHO). Thus, PDF characterizes the Doppler *E*-wave as the solution to Newton's Law for SHO motion, using three parameters: *k*, chamber stiffness; *c*, chamber relaxation/viscoelasticity; and *x*_o_, load. Most clinical *E*-waves are well fit by the “underdamped” (*c*^2^ < 4*k*) regime with solution for the velocity:

(A1)

The PDF parameters are calculated by using the *E*-wave contour as input. *E*-wave images are cropped, the maximum velocity envelope (MVE) is identified and used as input to fit the velocity of a damped oscillator using an iterative, error-minimizing Levenberg–Marquardt algorithm which has been described previously (Hall and Kovács 1994). The result solves the “inverse-problem of diastole” and provides unique PDF parameter values and the error of fit for each *E*-wave. The PDF parameters have invasive, gold-standard established physiologic analogues in terms of chamber stiffness, relaxation, and load (Kovács et al. 2000).

## Appendix B

### Equivalence of VFT standard expressions

The expression for VFT_standard_ used in this work (eq. 1) is equivalent to equation (B1) which is the expression used by Gharib et al. (2006). In this appendix we demonstrate that our expression for VFT_standard_ can be derived from Gharib's equation for VFT. The equation for VFT (which in Gharib et al. 2006 is referred to as T in eq. 1) is:

(B1)

In the above equation, EF is ejection fraction, *V*_A_ is the volume of blood filling during the A-wave, SV is the stroke volume, LVEDV is the left ventricular end-diastolic volume, and *D*_E_ is the effective orifice diameter. VFT can be rewritten as:

(B2)

Here, *V*_E_ is the *E*-wave volume. Given SV *=* EF·LVEDV and , where *U*_E_ is the mean *E*-wave velocity, t is the duration of *E*-wave (*E*_dur_). Substituting the value of *V*_E_ and SV into the expression for VFT.

(B3)

Hence, B1 and B3 are equivalent expressions and B3 is derived from B1. Assuming the *E*-wave shape as a triangle makes . Hence, we used B3 to calculate VFT_standard_.
